# Editorial Note: Tumor Necrosis Factor α-Converting Enzyme Inhibition Reverses Hepatic Steatosis and Improves Insulin Sensitivity Markers and Surgical Outcome in Mice

**DOI:** 10.1371/journal.pone.0362611

**Published:** 2026-11-12

**Authors:** The PLOS One Editors

The *PLOS One* Editors issue this Editorial Note to inform readers that concerns have been raised about results presented in Figs 3A and 3B (H&E panels).

The last author provided data supporting the results in [1] (S1 File). S1 File has not been editorially reviewed by PLOS, but it is provided here for readers’ consideration.

## Supporting information

S1 File Marimastat reversed hepatic steatosis in diet-induced obese mice as well as in *ob*/*ob* animals, as demonstrated by histology and magnetic resonance spectroscopy. C57BL/6 animals were fed a high fat diet for 9 weeks, followed by two weeks of treatment with either Marimastat (MAR) or vehicle (VEH) control. *Ob*/*ob* animals were fed a normal fat diet and received 4 weeks of MAR or VEH control. Representative liver sections stained with Periodic acid–Schiff (PAS; top panels; original magnification 200x), and Oil Red O (ORO; middle panels; original magnification 200x). Hepatic fat fraction was measured by magnetic resonance spectroscopy (MRS, bottom panels, representative animals). Percent fat content was determined relative to water (100%) by numerical integration of the areas under the lipid and water peaks. Livers from MAR treated diet-induced obese animals exhibited almost normal hepatic architecture, whereas VEH livers revealed micro- and macrovesicular steatosis (A). Livers from VEH treated *ob*/*ob* mice revealed extensive, predominantly macrovesicular steatosis (B). MAR treated livers from *ob*/*ob* mice, in contrast, showed a decrease in macrovesicular lipid droplets mainly around the portal tract (B). Histological findings were confirmed with MRS, demonstrating a decrease in fat content in Marimastat-treated animals (lower panels).
(PNG)
